# Using GIS to create synthetic disease outbreaks

**DOI:** 10.1186/1472-6947-7-4

**Published:** 2007-02-14

**Authors:** Rochelle E Watkins, Serryn Eagleson, Sam Beckett, Graeme Garner, Bert Veenendaal, Graeme Wright, Aileen J Plant

**Affiliations:** 1 Australian Biosecurity CRC, Division of Health Sciences, Curtin University of Technology, Perth, Australia; 2 Department of Spatial Sciences, Curtin University of Technology, Perth, Australia; 3 Office of the Chief Veterinary Officer, Australian Government Department of Agriculture, Fisheries and Forestry, Canberra, Australia

## Abstract

**Background:**

The ability to detect disease outbreaks in their early stages is a key component of efficient disease control and prevention. With the increased availability of electronic health-care data and spatio-temporal analysis techniques, there is great potential to develop algorithms to enable more effective disease surveillance. However, to ensure that the algorithms are effective they need to be evaluated. The objective of this research was to develop a transparent user-friendly method to simulate spatial-temporal disease outbreak data for outbreak detection algorithm evaluation.

A state-transition model which simulates disease outbreaks in daily time steps using specified disease-specific parameters was developed to model the spread of infectious diseases transmitted by person-to-person contact. The software was developed using the MapBasic programming language for the MapInfo Professional geographic information system environment.

**Results:**

The simulation model developed is a generalised and flexible model which utilises the underlying distribution of the population and incorporates patterns of disease spread that can be customised to represent a range of infectious diseases and geographic locations. This model provides a means to explore the ability of outbreak detection algorithms to detect a variety of events across a large number of stochastic replications where the influence of uncertainty can be controlled. The software also allows historical data which is free from known outbreaks to be combined with simulated outbreak data to produce files for algorithm performance assessment.

**Conclusion:**

This simulation model provides a flexible method to generate data which may be useful for the evaluation and comparison of outbreak detection algorithm performance.

## Background

Identifying disease outbreaks early is critical for efficient infectious disease control. Currently, spatial data are collected but often not well utilised in routine infectious disease surveillance. As outbreaks are often characterised by the degree of spatial diffusion of cases, spatio-temporal surveillance algorithms are being developed in a number of countries. These spatio-temporal algorithms aim to facilitate the early detection of disease outbreaks which exhibit spatial clustering [1], such as those associated with person-to-person transmission of disease, or a localised source of infection.

As work to develop spatio-temporal algorithms for the early detection of outbreaks of infectious disease continues, the importance of evaluating the performance of these algorithms increases. The evaluation process allows assessment of the timeliness and accuracy of algorithms in detecting defined disease outbreaks, and enables the selection of the most effective algorithm for each specific surveillance context [2]. The evaluation process also allows adjustment of the algorithm parameters to optimise performance for specific applications.

Both historical and simulated outbreak data can be used to evaluate algorithms for public health surveillance. Evaluation is typically performed by comparing algorithm-derived outbreak indicators with predetermined criteria indicating the specific location of outbreaks in time and space. The following section outlines the benefits and limitations of using historical and simulated data for the evaluation of outbreak detection algorithms.

### Historical data

Using historical data to evaluate outbreak detection algorithms ensures that the type of outbreaks used to test the algorithms reflects the properties of previous outbreaks. The extent of variability present in the evaluation data is also an important determinant of algorithm performance [2]. However, the precise occurrence of outbreaks in historical public health surveillance data is often not well-defined, and historical surveillance data generally contain few well-documented outbreaks which can be used to test algorithms. This produces limited opportunities to assess the timing and accuracy of outbreak detection. Historical data also often contain effects associated with changes in surveillance methods over time, including case definitions, which can produce apparent changes in disease incidence when no real change has occurred. These factors, alongside the presence of trends associated with changing disease epidemiology over time can limit the usefulness of historical data for the testing of outbreak detection algorithms.

### Simulated data

In contrast to historical data, evaluation of algorithms using simulated data allows more extensive evaluation of performance across a large number of datasets with precisely identifiable outbreak and non-outbreak periods. However, the form of the simulated outbreaks needs to be carefully considered, as the power of cluster detection methods to detect and locate disease clusters has been found to vary based on the location of the cluster, its extent, and the overall disease prevalence [3]. Furthermore, evaluations based on simulated data are limited by the extent to which the simulated data can adequately represent future surveillance reporting.

In recent years simulated data for benchmarking disease detection algorithms have been made widely available. For example, the Centres for Disease Control has developed 56 simulated datasets containing 1000 iterations of six years of daily data with ten different outbreaks randomly inserted [4]. Kulldorf et al. [5] also provide a large number of benchmark data sets for evaluation based on a random number of cases of a hypothetical disease. However, the format of benchmark datasets varies, and the majority of test datasets have been developed in the United States and are specific to this spatial context. This limits the usefulness of these data for the evaluation of outbreak detection algorithms designed for use in other settings.

Simulation software can also be used to generate data for evaluation. Two freely available programs have been designed for outbreak detection evaluation. The first is a temporal system known as HiFide [6]. HiFide inserts artificial spikes into time series data and provides excellent facilities for performance assessment. However, this approach is limited in that the program does not allow for spatial effects, and when evaluation is used to inform algorithm design, the use of a pre-defined outbreak shape will advantage algorithms that are best at detecting that particular outbreak shape [7].

The second program is the AEGIS Cluster Creation Tool which has also been recently developed for the creation of spatial-temporal disease outbreaks [8]. This open source program enables users to create simulated clusters and vary the desired cluster radius, density, distance, relative location from a reference point, and temporal growth pattern. Although this software provides a simple method for creating disease outbreaks, the outbreak generation methods are not designed to account for the mechanisms of spread of an infectious disease, including the underlying spatial distribution of susceptible individuals, and the resultant outbreaks are unlikely to provide a good indicator of the typical spatio-temporal arrangements found in historical disease data. As highlighted by Buckeridge and co-workers [7], many existing simulation approaches create signals with limited spatial complexity that do not incorporate knowledge of the disease being simulated nor explicitly model properties of the disease agent.

Other notable disease simulation tools exist [7,9-11], however, these have generally been designed to model data for specific diseases, locations or surveillance systems; or represent detailed models which are not easily applied to a new geography for which the models were not designed. For example, the BioWar simulator [10] uses a sophisticated model which is based on physician visit and pharmaceutical prescription data that may not be easily accessible in many other settings.

In summary, there are limited tools available that enable the spatial distribution of human disease outbreaks to be simulated based on general parameters. In this paper, we describe a simulation software application developed for use in evaluating outbreak detection algorithms for public health surveillance. The software simulates the occurrence of spatio-temporal disease outbreaks, accounts for the population distribution in the area under surveillance, and is based on a simple disease transmission model.

## Implementation

The objective of this research was to develop a transparent user-friendly method to simulate datasets for outbreak detection algorithm evaluation. To be effective for different locations the model uses the underlying population distribution and incorporates patterns of disease spread that can be customised to represent a range of infectious diseases. The software has been developed to model the spread of infectious diseases transmitted by person-to-person contact. An example application of the model for the South-west region of Western Australia is presented (see Additional file 1: MapBasic program files).

The primary output of the system is counts of cases of disease aggregated to postcode areas by day, which is the format currently used for surveillance reporting in Australia by the National Notifiable Disease Surveillance System (NNDSS), the main national public health infectious diseases surveillance dataset [12]. In the NNDSS, data are routinely collected nationally on approximately sixty different notifiable diseases and aggregated to the postcode of residence to preserve individual confidentiality.

The simulation software has been written and compiled in MapBasic version 8.0 (MapInfo Corporation, 2005) and runs within the MapInfo Professional Geographic Information System (GIS). MapBasic is a complete BASIC-like programming language for creating custom MapInfo Professional applications which have the potential to be integrated within Visual Basic, PowerBuilder, Delphi and C++ applications. The use of a GIS environment for the evaluation of disease outbreak algorithms allows the user to account for the underlying spatial distribution of the population, and enables access to spatially explicit functions. Working within a GIS also allows additional spatial datasets to be overlaid, and provides direct access to map and tabular outputs which can be used to monitor the impact of different variables on outbreak detection performance.

### Model approach

Using a stochastic simulation modelling approach allows outbreaks with a variety of characteristics that are epidemiologically plausible to be generated. This provides a powerful method to inform surveillance decision-making in the presence of uncertainty. The simulation modelling approach also permits the use of sensitivity analyses to explore the influence of uncertainty in estimated outbreak parameters and other data characteristics on algorithm detection performance. A similar approach has been used by veterinary epidemiologists to simulate outbreaks of foot and mouth disease [13].

**Figure 1:**
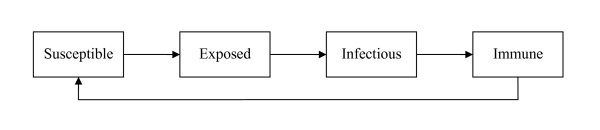
Model state transitions.

A stochastic state-transition model simulates disease spread in daily time steps using disease-specific infectivity and susceptibility parameters. The model tracks infection in individuals and is based on the SEIR (Susceptible, Exposed, Infectious, Recovered) approach commonly used to describe the epidemiology of infectious diseases [14,15]. The model records transitions from the initial non-diseased state, *susceptible*, in which individuals are susceptible to infection, to the *latent *(exposed) state, where an individual has been exposed to the infectious agent and has become infected, but is not yet able to infect others (Figure 1). The *infectious *state then follows once an infected individual is able to transmit the disease to others. Finally, the *immune *(recovered) state is entered once the individual has recovered from the infection and is then unable to be re-infected for a specified period of time.

The model is based on a proportionally sampled demographically homogenous population realistically dispersed in space. This can be achieved by using residential address points as a proxy for individual location data to provide a realistic indicator of population density. A demographically homogenous population provides a simplified model structure which is easily transferable between locations where detailed spatial demographic data are unavailable. The model can be extended to incorporate the population structure where these data are available. To limit the computing resources required for modelling very large or densely populated areas, an address point pre-processing routine can be used to randomly delete a specified proportion of address points within the study area.

**Figure 2:**
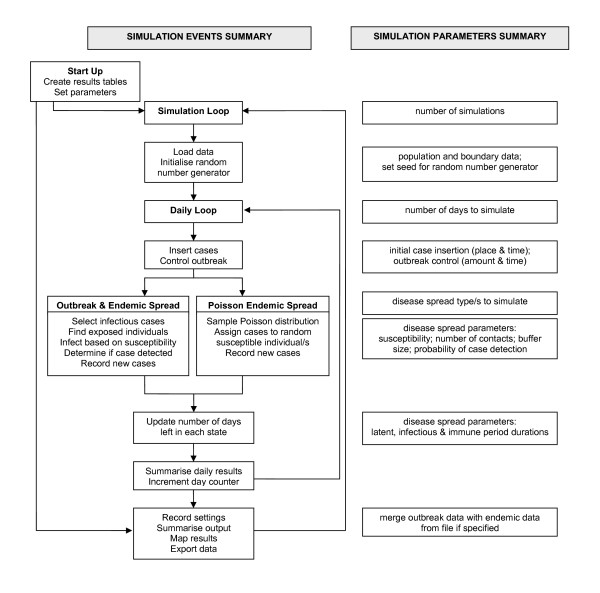
Process diagram of main events and adjustable parameters within the simulation.

Figure 2 illustrates the structure of the model and its daily loop for simulating infection processes. It also illustrates where in the modelling process the adjustable model parameters are used. The following section describes the key model parameters and events.

### Disease spread

The spread of disease is modelled according to two distinct theoretical components which together comprise the total observed cases of a disease at any time: an outbreak component, and an endemic component. The endemic component describes the variable and generally low level background component of disease which is commonly present in an area, and is not considered to be unusual. The outbreak component describes the component of disease which causes the observed incidence of disease to exceed the expected incidence (often in an epidemiologically significant subgroup), and this increase is considered to be of public health importance.

For many diseases there is no standard definition of the magnitude of increased incidence above expected levels that defines an outbreak, as the significance of an increase will vary according to a number of factors including the disease, setting, demographic characteristics, seasonal factors and factors related to the provision of health services which may influence the rate at which cases of disease are detected. Simulation methods are particularly appropriate for the evaluation of outbreak detection methods due to this variation in the characterisation of outbreaks.

The two components of observed disease incidence are modelled separately, such that individuals infected by the outbreak component of the disease incidence can only produce additional outbreak cases. Thus, the simulated outbreaks are generated from a distinct process, which occurs in addition to the normal background rate of disease that is not associated with factors responsible for the amplification in disease spread. This approach allows the calculation of key performance indicators of the timeliness and validity of outbreak detection algorithms based on the presence of the first outbreak case. The complete separation of outbreak and endemic disease processes is unlikely to represent the complex processes occurring in reality; however, it can describe scenarios where outbreaks are associated with differences in individual characteristics and behaviours such as food handling, personal hygiene, engaging in needle sharing, or being exposed to an infectious agent at a specific location, such as a restaurant.

#### Outbreak simulation

In the person-to-person disease spread model, variable distance circular buffers around infectious individuals are used to represent the likelihood of sufficient contact with susceptible individuals and the transmission of disease. This single distance-based summary exposure pathway models both the direct and indirect aspects of local disease spread, and represents epidemiologically significant spatial relations between individuals. Parameters can be altered to allow more or less clustering to reflect the importance of spatial proximity (as defined by residential address) in disease transmission.

The model can be extended to include additional disease spread pathways where appropriate to represent specific significant epidemiological linkages between individuals, such as school attendance or the impact of commuting behaviours. If sufficient data are available to allow parameterisation of specific disease spread pathways, incorporation of these into the model would require the use of additional variables and spatial functions to define the nature of the additional spatial contact patterns between individuals in the population. The parameters which determine whether an individual can transmit or acquire infection via specific disease spread pathways may also be associated with individual characteristics such as age, sex or employment status. Specific disease spread pathways are not used in the current model given our aim to create a generic model that requires few location-specific data sources. These could be incorporated for modelling particular disease scenarios when adequate data are available.

A general outbreak control process can be used to end the simulated outbreak. This process successively reduces the mean number of contacts each infectious individual has per day by a specified proportion, and can be theoretically related to the gradual implementation of disease control measures such as contact tracing, treatment and behavioural change. This parameter has been configured as a constant rate of decrease in the expected number of contacts each day, as the declining period of outbreaks is of limited interest for algorithm evaluation purposes, and the function improves the efficiency of the simulation.

#### Endemic simulation

The separate simulation of the endemic component of disease permits the use of both authentic and simulated endemic disease data in the model. The use of authentic endemic data in performance evaluations is recommended in guidelines for the evaluation of outbreak detection systems [2]. If authentic data are unavailable, endemic disease data can be simulated in the models using two methods. The first method uses the same person-to-person SEIR disease spread model as used for the outbreak spread pathway, but with different parameters, including large contact buffers, to produce a smaller number of cases which are not clustered in space. The difficulty with this method is that the parameters have to be carefully specified to produce low and relatively stable numbers of endemic disease cases, particularly over long time periods. The second technique available for simulating cases of endemic disease is similar to that used by Burkom and co-workers [16] to generate temporal background data using random draws from a Poisson distribution. We use daily random draws from a Poisson distribution to generate the number of cases of endemic disease occurring each day of the simulation, and each of the generated endemic disease cases is randomly allocated to a specific spatial location. This method provides a more reliable approach to simulating a Poisson spatio-temporal endemic case distribution than the person-to-person SEIR method. The method can be modified to incorporate other statistical distributions.

### Generalisability and uncertainty

The model is based on generic parameters to enable application across a variety of diseases that are spread by person-to-person contact, and a variety of contexts. The parameters of the model are generic in that they can be configured to reflect the properties of different infectious agents and different transmission characteristics. The parameters that determine disease spread are linked to underlying biological processes, and can be varied to produce outbreaks which are representative of historical data, based on known parameters such as latent and infectious period durations and geographic dispersion.

Uncertainty in the model, reflecting both parameter uncertainty and biological variability, is represented within the model through probability distributions, which are repeatedly sampled for each new event which occurs in the model, as has been used previously [17]. This approach allows sensitivity analyses to be conducted and linked to outbreak detection performance. Sampling processes within the model use the pseudo random number generator which is an in-built feature of MapBasic. The set seed function which is included in the main screen can be used to replicate random number sequences where required.

Beta-pert probability distributions are used in the current model to describe the time spent in each disease state. They only require specification of the minimum, most likely and maximum values for each distribution, and are well-suited to the parameterisation of expert opinion. The simulation model randomly selects a value from the specified distribution when a state transition occurs. A uniform probability distribution is used to select the buffer radius distance to identify potential close contacts for each infectious case for each day. The use of a uniform distribution also allows variability to be removed if desired by setting the upper limit of the distribution equal to the lower limit. Other probability distributions can be used in the model as required to better reflect biological variability and uncertainty associated with specific disease situations.

The number of close contacts that infectious individuals have each day within the eligible geographic area is randomly sampled from a specified Poisson distribution based on an expected small number of close contacts. However, based on previous empirical data, a normal distribution may be more appropriate for diseases when the mean number of significant contacts is expected to be large [18]. Random sampling using Monte Carlo methods is used within the model to select exposed individuals from eligible close contacts, to select infected individuals from among those exposed, and to select the location and time to seed the outbreak if desired.

This spatial simulation approach allows us to randomly simulate outbreaks in space and time and eliminate bias associated with the selection of the timing and location of outbreaks. The stochastic nature of the model ensures that simulations provide a representation of the possible range of outbreak scenarios based on existing knowledge about disease transmission. The distribution of outbreak characteristics such as the size and rate of increase in cases needs to be considered when comparing surveillance strategies. Sensitivity analyses should be used to explore the effects of recognised uncertainty in the specification of parameter values or distributions, and identify the variables which contribute to variation in simulated outbreaks and detection performance. Comparison of model outputs with public health surveillance data to verify that they reflect the range of outbreaks observed to date may be a useful means of validating the simulated datasets and model parameters.

Initial validation of the model included a comparison of the temporal and spatial profile of generated outbreaks of hepatitis A with available published data. Simulated outbreaks were generated which had temporal and spatial distributions which were similar to the observed historical data for outbreaks transmitted by person-to-person contact. Due to the relatively long latent period of hepatitis A, epidemic curves generally show an irregular rate of increase in case reports, often with a low number of case notifications early in the outbreak period (e.g. [19,20]), which was consistent with the simulation model output. Published reports also illustrate a high level of spatial clustering of Hepatitis A outbreaks (e.g. [19,21]), suggesting the local spread pathway may be reasonable in this context. Allard and co-workers [22] also highlight the proximity of cases which is associated with the clustering of place of residence according to risk factors for exposure. Further support for the validity of the simulation model was provided by the identification of recognisable epidemiological features of epidemic curves for propagated or progressive source outbreaks, being a series of taller peaks approximately one incubation period apart, in simulated outbreaks of varicella zoster virus based on published parameters [23].

### Running the program

**Figure 3:**
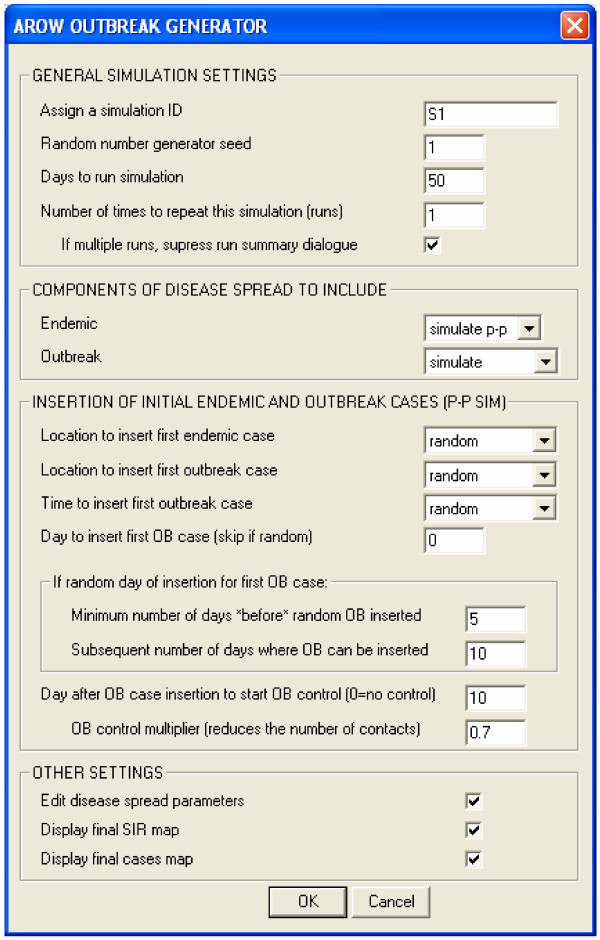
Start-up dialog.

**Figure 4:**
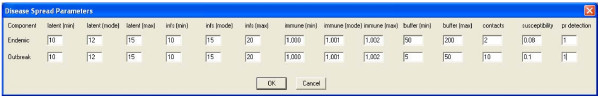
Disease transmission parameters dialog.

When the program is run, input screens are invoked (Figures 3 and 4) which allow parameters for the simulation to be specified. These include whether to simulate both the outbreak and endemic processes, and the option of inserting simulated outbreak data into an existing endemic data file. The simulation parameters, which can be randomly set or specified by the user, include the day to infect the first outbreak case and the location to insert the first outbreak case. The amount of time spent in each disease state, as well as individual susceptibility to infection (which determines the probability that an individual will become infected given exposure) are important determinants of the observed incidence of disease and are configured independently for the endemic and outbreak disease processes.

As the model is stochastic, the program is designed to perform multiple simulation runs – that is complete a simulation for say a 50-day period, and then repeat this process a number of times. Each time the 50-day simulation is repeated the program can be configured so that the initial cases are inserted in exactly the same place and time, limiting variation between runs to that produced by the intrinsic disease parameters alone, or for each run the timing and location of the initial cases can be selected randomly, producing both variation due to the disease parameters and variation due to differences in the timing and location of the increase in risk of disease.

Current temporal outbreak detection evaluation methods include testing using long outbreak-free datasets, and inserting a simulated outbreak at multiple points during this dataset to enable reporting of averaged detection abilities which account for typical fluctuations in baseline disease reports over time (e.g. [24]). The application of this technique in the spatio-temporal context produces a large number of potential day-location combinations for testing. The approach detailed in this paper uses random sampling over space and time across a large number of trials to account for the influence of space and time on performance. This random sampling process can be limited to urban areas, and other selection processes based on spatial or population attributes can be incorporated as required.

**Figure 5:**
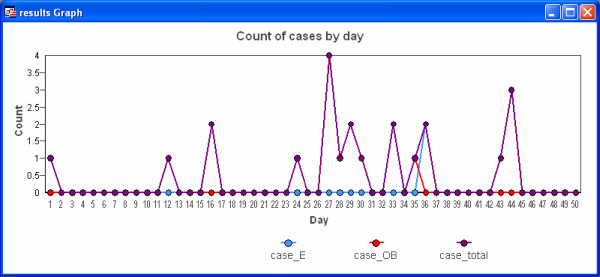
Time series graph of simulated outbreak cases in the South-west region of Western Australia.

**Figure 6:**
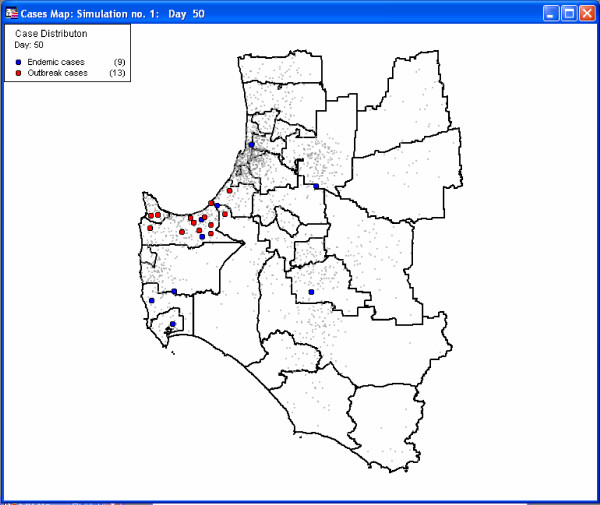
Map of simulated outbreak cases for the South-west region of Western Australia.

The software produces time series graphs of outbreak, endemic and total cases of disease by day of the simulation, as well as summary maps which illustrate the distribution of the population (light grey dots) and highlight the location of cases. Summary data are stored in tables. Figures 5 and 6 present example graph and map outputs from a 50-day simulation for the South-west region of Western Australia that included both outbreak and endemic disease components inserted at random locations. The first outbreak case was infected randomly between the 5^th ^and 15^th ^days, with outbreak control commencing 10 days following this date. The parameters for endemic spread (Figure 4) were chosen to produce a lower rate of disease transmission.

### Flexibility

Few modifications are required to operate the existing model with new location data. The user is required to either obtain or generate a representative population sample of individuals as point-level data. Address points provide a convenient source, although other datasets which incorporate information about spatial location of the underlying population could be used. The simulation program also requires a second file containing the boundaries of the spatial areas for aggregation and reporting purposes – for example postcode boundaries. The unique identifier for these small areas also needs to be included in a single-column table, which is used to record the results of the simulation by area and day. If simulated outbreak data are to be inserted into an existing endemic data file, this data file also needs to be imported into the project.

## Simulation example

An example simulation of a large outbreak of influenza is used to illustrate the application of the model for outbreak detection evaluation. The simulation was designed to provide data to estimate the expected average performance of an outbreak detection algorithm for Western Australia based on daily notifiable disease surveillance, given the specified outbreak scenario. A 10 per cent random sample of address points was used to represent the population distribution of Western Australia (165165 individuals), and postcode boundaries were used for summary purposes, as these are the spatial units used for disease reporting nationally in Australia. We simulated endemic disease data using the Poisson endemic method with a reasonably low average case rate of 0.5 cases per day to avoid using confidential endemic data. However, we recommend using authentic endemic data whenever possible to provide a more realistic indicator of the background noise that will affect outbreak detection performance.

The simulation was run for a period of 50 days, which included an initial period of 10 days where only endemic cases were simulated to ensure that the initial algorithm analysis period (e.g. a 7-day window) did not include the outbreak insertion date. The time of outbreak insertion was allowed to vary randomly between day 10 and day 40, which allows sufficient time for an outbreak to develop and be detectable before the end of the simulation. In practice, longer simulation periods would be selected to provide a potentially larger degree of variation in endemic disease activity which may influence outbreak detection performance.

As we aimed to simulate a highly clustered outbreak, a minimum buffer width for disease transmission of 5 km and a maximum buffer width of 10 km were used. The parameters for the latent and infectious periods were based on those which describe the natural history of the 1957–58 pandemic influenza A virus [25], a particularly severe strain. Beta-pert distribution parameters of (0.5,1.9,3.5) and (2.5,4.0,6.5) were used to model the latent and infectious periods and approximate the parameters used previously [25]. The immune period duration was set to an arbitrary mean which exceeded the length of the simulation period to prevent cases from being a re-infected within the simulation period.

**Figure 7:**
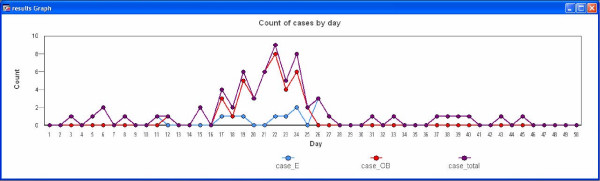
Time series graph of a simulated influenza outbreak in Western Australia.

For the current analysis we assumed that the number of contacts of cases was Poisson distributed with a mean of 2 persons per day, and that 33 per cent of the simulation population were susceptible to infection, which is equivalent to the average attack rate reported by Longini et al [25]. Susceptibility to infection can be adjusted to reflect different levels of natural or acquired immunity to produce outbreaks of various sizes. As the initial outbreak period was of interest, outbreak control was commenced 10 days following insertion of the first case (day 12), which results in daily incremental reduction of contacts and elimination of the outbreak by day 26 (Figure 7). Outbreak control processes can be introduced earlier to reduce simulation time.

The 50-day simulation took 35 minutes to simulate a total of 128 exposures which resulted in a total of 25 endemic and 41 outbreak cases using a laptop computer with a 1.6 GHz processor and 512 MB of RAM. To illustrate the significant impact of population size on simulation completion time, the same parameters were used to simulate a 100-day uncontrolled outbreak using a population size of 1921 individuals. This took 8 minutes to simulate a total of 19720 exposures resulting in a total of 48 endemic and 1683 outbreak cases using the same computer.

Graphical outputs from the model include a time series graph which displays counts of simulated cases by day that provides an indicator of the progress and outcomes of the simulation (Figure 7), and maps of outbreak and endemic cases are produced at the conclusion of the simulation. The model was found to produce a typical epidemic curve for a large outbreak of influenza. Data outputs include line listings of cases and exposures by day of exposure or infection, and summary of the data as counts of cases by day and postcode which can be analysed by outbreak detection algorithms. When authentic endemic data is used in the simulation, an additional output file identifies only the outbreak cases, which can be used to assist in automating the identification of valid outbreak detection days and areas in performance analyses.

As little is known about the processes associated with care seeking and rates of case detection for influenza, the parameterisation of care-seeking processes is difficult. The simulation scenario described assumed a 100% case detection rate, and that case detection occurs following the transition to the infectious state. Sensitivity analyses can be used to vary the case detection rate and estimate the impact of case detection on outbreak detection performance. Depending on the disease studied and processes of interest, alternative representations of disease states can be used, including renaming the four disease states used here, or including additional states to represent the presence of clinical symptoms.

The software does not itself assess detection performance. Thus, there are no constraints on the outbreak detection performance measures that can be applied to the model outputs. Performance indicators including timeliness and false alarm rates can be applied temporally or spatio-temporally to assess whether alarms occur during a defined outbreak period and in an outbreak-related location. Timeliness can be related to the time since the first outbreak case or to the cumulative number of outbreak cases which have occurred, which may be more intuitive when describing the timeliness of detection performance for diseases with long latent periods. These performance indicators can then be averaged based on many simulations and compared for outbreaks of various sizes (by adjusting the susceptibility or contact parameters) for outbreaks with different degrees of clustering (by adjusting buffer settings) and for different levels or sources of endemic disease data.

## Results and discussion

This simulation model provides a flexible method for generating datasets and selecting an unbiased sample of days and locations to insert outbreaks for the evaluation of spatio-temporal outbreak detection algorithms. This enables the selection of locations sampled to be weighted according to population density. It also allows the use of historical data in evaluation, as it is important that algorithms be robust to variations present in real data [9].

Models are necessarily idealised, and like others [7] the scope of the model has been limited by focussing on the evaluation of timely outbreak detection in a generic context. The model focuses on the effects of population density and spatial distribution, and agent-based epidemiologic characteristics influencing disease spread, including susceptibility, transmission rates, and the duration of latent, infective and immune periods. Others have also used general disease characteristics such as the temporal profile of the epidemic curve and short or long incubation times [4] to simulate outbreaks which are not disease specific. These simulation approaches are well suited for the evaluation of methods that are designed to detect outbreaks across a variety of situations and data sources.

This model provides a method to explore the ability of outbreak detection algorithms to detect a variety of events across a large number of stochastic replications where the influence of uncertainty can be controlled. These features are essential to address the main limitation associated with using simulated data for evaluation, which is that the data do not represent real outbreaks and may not provide a good representation of future outbreaks. Simulated data can be compared with historical data and records of events of public health importance to support their validity, and can be analysed by the parameters used to account for variations in performance. The current approach provides a simplified model for generating endemic cases of disease. As such, we recommend the use of historical endemic disease data for algorithm evaluation where these are available.

The current simulation model is simplified in order to maintain a straightforward model structure and avoid introducing unnecessary artefacts of the simulation process into the evaluation datasets, which may bias the evaluation findings. We acknowledge that even simple simulation models may introduce unwanted effects into the evaluation datasets, as they simplify the complex process of outbreak evolution.

The current simulation model also does not account for effects associated with individual health seeking behaviours, health service provision, and the intricacies of diagnosis and reporting of disease to health authorities. The factors influencing diagnosis and reporting are likely to be highly context and disease dependent, and require additional data to model accurately. Recent research has found that the effects of modelling the proportion of people seeking care were similar to the effects observed when the size of the outbreak was decreased [9]. This finding suggests the use of a simplified approach, which excludes explicit modelling of individual diagnosis and reporting issues when insufficient data are available, may be adequate.

The state-transition approach implemented in the model currently links the detection or reporting of cases of disease with the transition of the individual into the infective state. If the timing of the detection of disease needs to be represented as distinct from the time of transition to the infectious state, then additional disease states can be easily incorporated into the model. For example, a separate 'symptomatic' state may be required for diseases where individuals are infectious for a significant period prior to becoming symptomatic. The model is also configured to produce area-level output data, as analyses of health data often use aggregated data to protect individual confidentiality. However, as the underlying model is based on individual locations, other output options such as point-level data can be produced.

### Future development

In developing this simulation model the aim was to restrict complexity and produce a flexible model that has the ability to represent disease transmission and its spatial distribution for different diseases in a variety of settings. The simple structure of the single disease spread pathway used in the current model allows transparency of operation and simplifies the influence of uncertainty with respect to disease transmission. Initial results indicate that this approach can provide an adequate representation of outbreaks for diseases which are predominantly spread by person-to-person contact or temporary local environmental contamination. Future work will examine specific exposure pathways, demographic heterogeneity, and changing behaviours following the onset of illness.

The use of a GIS platform makes it possible to readily incorporate further spatial complexity in the model, including risk factors associated with specific locations and additional disease spread pathways. Additionally, the model can be extended to include demographic heterogeneity in differential individual susceptibility, infectiousness or behaviour when this information is available. By using address point data, the modelling of individuals within households using available group-level data can be used to overcome the potential under-representation of populations in some areas associated with allocating individuals proportional to households. However, this is not likely to be a significant concern given the relatively rare nature of the infectious diseases for which the system was designed, and the aggregation of data to the postcode level.

The validation of a disease outbreak simulation model is challenging given the poorly defined and variable nature of outbreaks. Few published studies that have used simulated data for the evaluation of outbreak detection methods have evaluated the validity of the simulations in any formal manner. Simulated data are commonly acknowledged to be approximations which have been generated based on previous data (e.g. [4]). Strategies used to support validity have included the incorporation of uncertainty using sensitivity analyses, modelling a range of possible scenarios [9], and simulating a range of outbreaks so that those most similar to observed outbreaks for the disease under investigation can be used [4]. Future studies are planned to validate the model across a range of diseases.

## Conclusion

The aim of this project was to create a method for simulating disease outbreaks which can produce realistic case distributions, and be easily adapted for different locations and to represent different underlying population distributions in order to evaluate outbreak detection algorithms. The approach described facilitates the comparison of algorithms for early detection within a spatially relevant context. It is envisaged that the software will be developed further to include more detailed consideration of modes of disease spread, and to incorporate case detection and disease control processes. The implementation of the simulation model in a GIS environment, although not the most efficient approach, provides significant advantages including access to a large number of spatial functions, and is suited to the rapid development and prototyping of spatially explicit simulation models.

## Availability and requirements

Project name: none

Project home page: none

Operating system: Windows

Programming language: MapBasic

Other requirements: MapInfo Professional

License: none

Any restrictions to use by non-academics: none

## Competing interests

The authors declare that they have no competing interests.

## Authors' contributions

REW and SE conceived and designed the study and conducted the literature review, REW, SE, SB and GG contributed to software and model design and construction, REW and SE were involved in drafting the manuscript, and REW, SE, SB, GG, BV, GW and AJP were involved in critically revising the manuscript. All authors read and approved the final manuscript.

## Pre-publication history

The pre-publication history for this paper can be accessed here:



## Supplementary Material

Additional File 1 **MapBasic program files**. This archive contains all the files required to run the software if MapInfo Professional is installed. The OutbreakSim folder must be placed within the MapInfo folder. Click here for file
