# Supplementary figures and images for: Using GIS to create synthetic disease outbreaks
Source: BMC Med Inform Decis Mak. 2007 Feb 14;7:4. doi: 10.1186/1472-6947-7-4 (PMC1805744; doi:10.1186/1472-6947-7-4)

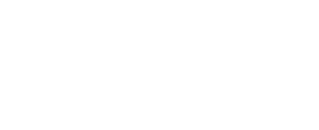

Supplement: Additional File 1 — MapBasic program files. This archive contains all the files required to run the software if MapInfo Professional is installed. The OutbreakSim folder must be placed within the MapInfo folder. [file 1472-6947-7-4-S1.zip › OutbreakSim/blank.bmp]
